# The Best Form of Medical Service

**Published:** 1921-02-26

**Authors:** 


### THE EDITOR'S LETTER-BOX.

Correspondence on all subjects is invited, but we cannot in any way be responsible for the opinions expressed by our correspondents, who must give their name and address as a guarantee of good faith, but not necessarily for publication. Correspondents are reminded that brevity of style and conciseness of statement greatly facilitate early insertion.

### The Best Form of Medical Service.

*To the Editor of* The Hospital.

Sir,—From what I have read in the newspapers I have certainly come to think that Dr. Addison's aim is to have the entire medical service of the nation State-controlled—that we should all be "panel" patients. From the point of view of not only the so-called working classes but the poorer middle classes, it would be by no means a bad thing to know exactly what one's doctor's bill would be for the year, as one does one's fire insurance. Some years, doubtless, we should not get value for our money, but other years we should get more. And as the doctor would make no bad debts he would probably be reasonable in his demands. But it is essential that there should be a free choice of doctor. The panel system has been in many cases one of the least-pleasing features of the existing Health Insurance Act. Many working people, even though they pay the insurance, go to a private practitioner because they think, rightly or wrongly, that the panel doctor gives them but scant attention. If when the Act was passed every doctor had been permitted to retain his poorer patients on a basis of annual payment at insurance rates, most would have done so, old ties—often very kindly ones—would have been maintained, and the patients would certainly have been more content. On the one hand, the limitation of choice, so hateful to the British temperament, caused a sense of grievance, while many doctors who would have been quite willing to keep on the humbler patients whom they knew, felt that they would lose status and perhaps have more work thrust on them than they could conscientiously undertake if they became panel doctors. These considerations were ignored in the Health Insurance Act, but in matters of sickness and health the human element is all-important, and certainly it ought to be taken into consideration in any extension of the Act to include the middle classes. English people take kindly to the idea of insurance, but they like to choose their own office. Given a free choice of doctor I think it would be possible to arrange for insured medical treatment for households where the family income does not exceed £500 a year. But free choice is essential.—Yours, &c.,

Middle-class Widow.

